# Outcomes following Management of Complex Thoracoabdominal Aneurysm by an Open Approach

**DOI:** 10.3390/jcm12093193

**Published:** 2023-04-29

**Authors:** Roberto Chiesa, Enrico Rinaldi, Andrea Kahlberg, Sarah Tinaglia, Annarita Santoro, Giovanni Colacchio, Germano Melissano

**Affiliations:** 1 Division of Vascular Surgery, IRCCS San Raffaele Scientific Institute, Vita-Salute San Raffaele University, Via Olgettina, 60, 20132 Milan, Italy; 2 Department of Vascular and Endovascular Surgery, General Regional Hospital Ente Ecclesiastico “F. Miulli”, 70021 Acquaviva delle Fonti, Italy

**Keywords:** aorta, thoracoabdominal aortic aneurysm, TAAA, open surgical repair

## Abstract

Background: In the last decade, advances in surgical techniques, and the introduction of adjuncts for organ protection, have modified the approach for thoracoabdominal aortic aneurysm (TAAA) surgical repair. The aim of this study is to determine whether the contemporary approach influenced the outcomes. Methods: From 1989 to 2022, patients who had received elective open surgical repair (OSR) for TAAA at our institution were retrospectively analyzed. This series has been divided in two groups: Group 1 (1989–2009), and Group 2 (2010–2022). Patients included in Group 1 were those treated with a selective use of adjuncts, and Group 2 included patients treated with the systematic use of adjuncts. Results: A total of 1107 patients were treated (Group 1: 455; Group 2: 652). The surgical management was significantly different between the two groups. The in-hospital mortality was significantly different between the two groups (Group 1: 13.4%, Group 2: 8.1%; *p* 0.004), as was the rate of permanent spinal cord ischemia (Group 1: 11.9%, Group 2: 7.8%; *p* 0.023). Renal and respiratory failure were reduced in Group 2, but not significantly. Conclusions: The use of the adjuncts enabled the achievement of improvement in mortality and SCI prevention in TAAA OSR. Although a refined surgical technique, mortality and morbidity are still noteworthy in this complex aortic field.

## 1. Introduction

Thoracoabdominal aortic aneurysm (TAAA) open surgical repair (OSR) is one of the most extensive procedures performed in vascular surgery, and is a formidable challenge for surgeons, anesthesiologists and patients alike. This procedure, generally performed in elderly patients afflicted by multiple comorbidities, owing to both the technical difficulties of aortic reconstruction and the organ stress suffered. As a result, TAAA OSR is associated with not negligible mortality and morbidity rates [1,2,3,4,5]. In the recent past, the mortality rate after TAAA OSR has been reported between 2.3% and 32.7%, with better results reported in high-volume centers. However, in the last decade, advances in surgical techniques and in organ protection methods have led to considerably better outcomes [1,3,4,6]. 

In parallel with the advancements in the OSR technique, endovascular techniques have emerged as an appealing alternative for the treatment of the thoracoabdominal diseases, with encouraging results [3]. In this scenario, it is good timing for a re-evaluation of the safety of the OSR. 

In this study, we report an analysis of our experience in TAAA OSR, together with a description of the evolution of our therapeutic approach. This experience has influenced our treatment protocol and improved our results.

## 2. Materials and Methods

### 2.1. Patients

Patients who had received OSR for TAAA at our institution between January 1989 and December 2022 were prospectively included in a database and retrospectively analyzed. Clinical data from patients with TAAA were analyzed for preoperative characteristics and comorbidities, intraoperative details, and outcomes. Patients with contained ruptured or clearly ruptured TAAA were excluded from the analysis.

During this 33-year experience, the preoperative, intraoperative, and postoperative management of patients with TAAA, treated by means of OSR, has changed, with the introduction of several modifications. These adjunctive maneuvers and technologies, grouped under the name of “adjuncts”, have been progressively introduced into the clinical practice, initially in selected cases, and then routinely. In order to evaluate the role of these adjuncts, and their impact on the postoperative results, the overall experience has been divided in two consecutive groups: Group 1 (from January 1989 to December 2009), and Group 2 (from January 2010 to December 2022). Thus, in Group 1, patients from the early experience treated with a selective use of some adjuncts were included, while, in Group 2, patients from the more recent experience were included, treated with the systematic use of all the currently available adjuncts.

Patients gave written informed consent for the anonymous collection of their data for the study in the consent form provided by our institution and approved by our Institutional Review Board, in accordance with Italian law.

### 2.2. Patient Evaluation, Imaging and Preoperative Adjuncts

Our routine preoperative screening includes an evaluation of the carotid and peripheral artery axis by means of color-coded duplex Doppler. Lung function is tested by means of chest X-rays, blood gas analysis, and respiratory functionality tests. When severe chronic obstructive pulmonary disease (COPD) is present, and the patients’ general and anatomical conditions are adequate, home respiratory therapy, physiotherapy, and correction of risk factors (smoking cessation), are carried out by the patients, eventually also with the use of bronchodilator drugs and cortisone. Renal function is tested with blood tests and chronic renal failure is defined as an estimated glomerular filtration rate < 60 mL/minute/1.73 m^2^. 

Provided that there are no specific contra-indications, the preferred imaging modality used at our center is multidetector computed angiography tomography (CTA) scanning. New and updated postprocessing software have been introduced in order to allow a complete evaluation of the images, including useful measuring tools such as multiplanar reconstruction, surface and volume rendering. A preoperative, thorough evaluation of the CTA with the postprocessing software allows a complete analysis of the aorta, the collateral vessels, and also of the spinal cord (SC) vasculature [7]. Patients with aortic aneurysms are frequently affected by coronary artery disease (CAD), and since 2010, a preoperative ECG-gated coronary computed tomography angiography (CoroCTA) has been introduced as part of the preoperative work-up in patients with TAAA [8]. With this examination, it is possible to assess, at the same time, both the aorta and the coronary arteries. Thus, with CoroCTA, it is possible to identify concomitant coronary stenosis or occlusion, in order to better stratify the patient’s risk, and with the possibility of treating the patients for CAD before the TAAA OSR. When significant flow-limiting CAD is detected, a preoperative percutaneous coronary angioplasty with bare metal stent is generally preferred over other approaches [9].

### 2.3. Patient Preparation, Surgical Technique, Intra- and Postoperative Adjuncts

Spinal cord ischemia (SCI) is one of the most dreadful complications in patients who undergo TAAA OSR, and it may affect both perioperative and long-term outcomes because of multiple and severe medical complications directly related to the neurologic damage [10,11,12,13]. Cerebrospinal fluid drainage (CSFD) is an adjunct that has been proven to be effective in SCI prevention in TAAA OSR in randomized controlled trials and meta-analyses, and its usage is also suggested by the most recent guidelines [14,15]. In our practice, CSFD is placed in the operatory room by the anesthesiologist before the general anesthesia is induced; an epidural catheter is usually placed at the T7–T8 level using a loss-of-resistance technique. CSFD was introduced in our clinical practice in 2003, and for ten years, it was performed using dripping chamber-based systems; in 2013, an automated system for CSFD measurement and drainage (LiquoGuard-Möller Medical GmbH, Fulda, Germany) was introduced, and it replaced the previous manual system [16]. In our practice, CSFD is performed in all the patients with TAAA extension I to III, according to Crawford’s classification (without specific contraindications; e.g., tonsillar sagging), and selectively in patients with extent IV TAAA (considered at high risk for SCI; e.g., hypogastric artery occlusion, subclavian artery occlusion) [17]. CSFD is generally maintained for 48–72 h after surgery, or more, in the case of neurological impairment. After CSFD placement, the neurophysiologist arranges the electrodes for the intraoperative neurophysiologic monitoring. In 2012, we introduced the routine monitoring of motor and somatosensory evoked potentials (MEP&SSEP) during TAAA OSR for early detection of possible spinal cord impairment during the procedure [18]. 

After the preoperative maneuvers, and the patient positioning, the surgical procedure is initiated. The surgical technique used for TAAA OSR at our institution has been previously described [19]. Briefly, after thoracic and abdominal aortic exposure, the left superior (or inferior) pulmonary vein (LPV), and the left common femoral artery (CFA), are exposed for left heart bypass (LHBP). A cannula is introduced in the LPV, and the oxygenated blood is drained and then re-infused through a centrifugal pump into the left CFA for retrograde perfusion of intercostal, visceral, and renal vessels during sequential cross-clamping maneuvers. Two perfusion catheters are also connected to the circuit and used during visceral aortic replacement for celiac trunk (CT) and superior mesenteric artery (SMA) selective perfusion. The LHBP was introduced in our practice in 1993, and it is used at our center in all the patients with TAAA extension I–III, and selectively in patients with extent IV TAAA (considered at high risk for SCI). In addition, since 2013, the entire surgical procedure has been performed under continuous trans-esophageal echocardiography (TEE), which allows an early detection of any cardiac function impairment, and offers the possibility to apply prompt modifications to modulate the cardiac afterload. 

**Figure 1:**
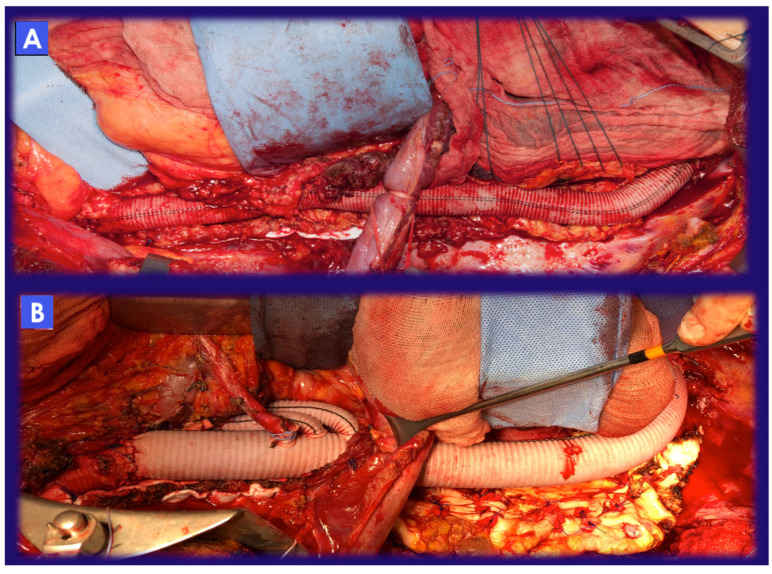
(**A**) Final reconstruction after extent II TAAA OSR with a tube graft. Visceral and renal vessels have been reattached by means of a Carrel patch (a single aortic island with the inclusion of visceral and renal ostia). (**B**) Final reconstruction after extent II TAAA OSR with a branched graft. Visceral and renal vessels have been reattached by means of selective bypasses.

After the LHBP is started, the thoracic aorta is gently sequentially cross-clamped: the proximal clamp is placed at the most convenient site, usually after the left subclavian artery (LSA), or between the left common carotid artery (LCCA) and LSA; the distal clamp is placed few centimeters below the proximal one. The descending thoracic aorta is then transected and separated from the esophagus, and non-critical intercostal arteries are ligated. The proximal end of the graft is sutured to the descending thoracic aorta using a running suture reinforced with felt pledgets. The clamp is then removed and applied onto the distal thoracic aorta above the celiac axis; the aortotomy is then extended to the diaphragm and the intercostal arteries are identified and temporarily occluded. The reattachment of critical patent intercostal arteries (from T7 to L2) to the aortic graft may play a critical role in spinal cord protection [20]. In this phase, MEP&SSEP are used to detect any spinal cord perfusion impairment, and intercostal arteries are immediately reattached to the aortic graft in the case of MEP&SSEP alterations. Intercostal arteries may be reattached using a tailored side-cut in the graft, by means of an island technique, or with selective bypasses. The distal clamp is moved below the renal arteries, the aneurysm is opened, and CT and SMA are catheterized and selectively perfused with blood from the LHBP. Renal arteries are catheterized as well and perfused with a cold solution; since 2009, a selective renal perfusion with histidine-tryptophan-ketoglutarate (HTK) solution (Bretschneider’s) at 4 °C has been performed during TAAA OSR for renal protection [21]. Visceral and renal arteries are reattached to the aortic graft by means of a tailored side cut in the graft (Carrel patch), or with selective bypasses. In patients with generically triggered aortic diseases (e.g., Marfan syndrome), the reattachment of large amounts of native aorta is generally avoided in order to prevent possible late dilatations of the remnant aortic tissue, and the aortic replacement is preferably performed using branched surgical grafts (Figure 1) [22]. 

Finally, an end-to-end anastomosis with the distal aorta is performed and the last clamp removed. A specific surgical approach, with avoidance of sequential aortic cross-clamping, was adopted in the case of extensive aortic wall thrombosis in order to reduce the risk of thrombus mobilization and embolization [23]. To reduce the postoperative thoracic pain associated with the invasive surgical access needed for TAAA OSR, since 2021, intraoperative intercostal nerve cryo-analgesia has been introduced in our standard practice [24]. 

Despite the surgical experience, TAAA OSR may cause high rates of intra- and perioperative bleeding, and the need for blood product transfusions, also considering that the loss of large amounts of blood not promptly replaced is associated with hypotension and may be an adjunctive risk for SCI and organ hypoperfusion in general. On the other hand, blood transfusion may be associated with well-known complications and lung injury; thus, management of bleeding is considered of paramount importance in TAAA OSR [25]. The rotational thromboelastometry (ROTEM) is able to detect specific alterations in the coagulation cascade early, and it was introduced in our practice in 2016. With this tool, it is possible to detect isolated deficits of fibrinogen and abnormalities of the intrinsic and extrinsic pathway of the cascade. Furthermore, APTEM and HEPTEM provide information, respectively, on hyperfibrinolysis and the presence of heparin in the patient’s blood. Alterations may be treated with the possibility of reducing intraoperative bleeding and the amount of transfusions [26]. After surgery, patients are kept sedated and intubated and transferred to the ICU. In general, weaning from mechanical ventilation is started the morning after surgery if patients achieve normothermia and hemodynamic stability, and have regained full consciousness. After 1 h on pressure support ventilation, extubation is performed if arterial blood gas analysis shows a PaO2/FiO2 > 200, normocapnia, and a pH within normal ranges. Selective non-invasive ventilation (NIV) during the postoperative period has shown positive results in terms of respiratory insufficiency prevention in patients with TAAA treated by means of OSR in our experience. For this reason, since 2014, early prophylactic NIV has been performed in all the patients (without specific contraindications, e.g., emphysema bubbles) after surgery [27]. 

**Figure 2:**
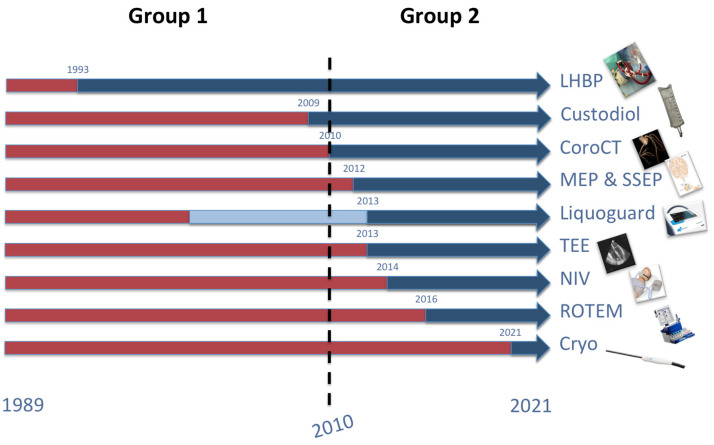
Graphical summary of the different adjuncts introduced in our clinical practice, and their time of introduction. Figure legend: red color stands for no use of the adjuncts; blue color stands for the use of the adjuncts. For CSFD, red color stands for no use of CSFD; light blue stands for the use of dripping chamber-based systems; blue color stands for the use of the Liquoguard.

All the adjuncts used during TAAA OSR, and their times of introduction are summarized in Figure 2.

### 2.4. Outcomes

As primary endpoints were considered the perioperative outcomes, defined as occurring within the first 30 days after surgery or during the hospital stay if >30 days, and included: 30-day mortality, respiratory failure (defined as need of prolonged intubation with >48 h after surgery, need of re-intubation or tracheostomy), permanent SCI, renal failure (defined as acute kidney injury with loss of function or end stage renal disease, stage 4 or 5, according to the risk, injury, failure, loss of kidney function, and end-stage kidney disease (RIFLE) classification), myocardial ischemia, major stroke, mesenteric ischemia (with need of intervention), and need of re-intervention (including any kind of re-intervention within the hospital stay) [28]. During the postoperative period, patients developing symptoms of SCI were evaluated by a neurologist to confirm the diagnosis and assign a grading; permanent SCI was reported in the case of permanent deficit, both minor and major. NIV was not considered as an indicator of respiratory insufficiency due to its prophylactic use during the postoperative period at our center. 

### 2.5. Statistical Analysis

Descriptive statistical analysis was performed using numbers and percentages for discrete variables, mean ± standard deviation for continuous variables, and median and interquartile range for variables with a non-normal distribution. Continuous variables were compared by the Student’s *t*-test; categorical data were analyzed using the chi-square test. Statistical significance was defined as a *p*-value < 0.05. All analyses were performed with the Statistical Package for Social Sciences software (SPSS Inc, Chicago, IL, USA), version 26.0 for MacOS. 

## 3. Results

From 1989 to 2022, a total of 1107 patients underwent elective TAAA OSR at our institution. The OSR was performed for TAAA I–III extensions in 80.8% of the cases, mainly for degenerative etiology. The entire cohort was divided in two groups; 455 patients were included in Group 1, while 652 patients were included in Group 2. The two groups were similar for the majority of the preoperative characteristics, except for smoking habits and history of aortic surgery; patients in Group 1 had a significantly higher rate of history of smoking (*p* 0.021), but the two groups were similar for preoperative COPD. A significantly higher rate of TAAA OSR was performed in patients with previous aortic surgery (*p* 0.033) in Group 2. Demographics and baseline characteristics of both groups are summarized in Table 1. 

**Figure 3:**
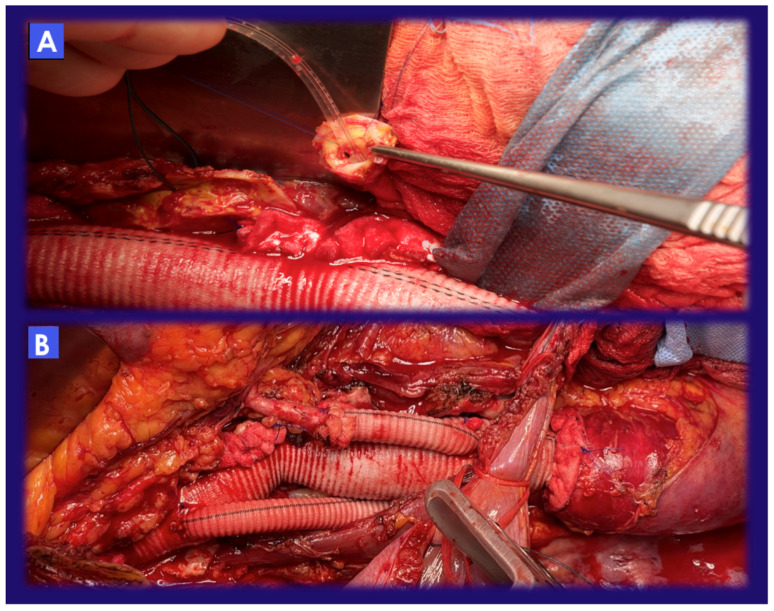
(**A**) Catheterization and perfusion of the left renal artery with an occlusion–perfusion Pruitt catheter; the left kidney was perfused with the HTK solution. (**B**) In order to reduce the size of the Carrel patch, the left renal artery was divided from the aorta and reattached by means of a selective bypass from the aortic graft. In this picture is also possible to recognize a selective bypass for intercostal arteries.

**Figure 4:**
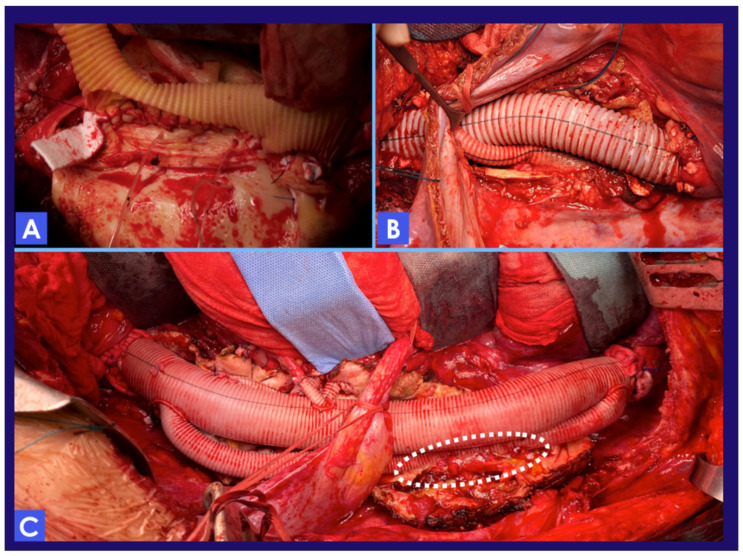
Intercostal artery reattachment with three different techniques. (**A**) An aortic island including the origin of several intercostal arteries is reattached to a fenestration created on the aortic graft. (**B**) Intercostal arteries are reattached selectively to the graft via 6/8 mm interposition grafts. (**C**) Another possible way to reattach critical intercostal arteries is represented by the “loop graft”. A 14/16 mm graft is anastomosed proximally and distally to the aortic graft. A fenestration is created in this loop graft to reattach the origin of multiple intercostal arteries (dotted circle).

The two groups were significantly different for what concern the use of the intraprocedural adjuncts, and for many aspects of the surgical procedure. Namely, CSFD, TEE, MEP&SSEP, LHBP, renal perfusion with HTK solution, and ROTEM were employed routinely in Group 2, with statistically significant differences if compared with Group 1. The surgical procedure was also different in patients in Group 2, concerning the site of aortic clamping, the technique used for the aortic replacement and the visceral vessel reconstruction, and the rate of intercostal artery reattachment. We observed a higher incidence of “high” proximal aortic cross clamping between the LCCA and the LSA, in Group 2. Furthermore, the technique for visceral and renal vessel reconstruction was significantly different between the two groups: while in Group 1, a Carrel patch (or beveled) with the inclusion of CT, SMA, and both the renal arteries was the preferred approach (57.4%), in Group 2 smaller patches associated with selective bypasses were preferred (59.7%) (Figure 3). Lastly, in Group 2, intercostal arteries were reattached to the aortic graft more aggressively (*p* = 0.044). Different techniques have been used for intercostal artery reattachment, such as aortic island, selective bypass, or loop bypass (Figure 4). Intraprocedural details and the usage of intraoperative adjuncts are summarized in Table 2. 

The overall 30-day mortality was 10.3% (114/1107); the causes of death were multiple organ failure (MOF) in 44 cases, cardiac complications (including myocardial ischemia and arrhythmia) in 36 cases, bleeding in 12 cases, systemic embolization in 14 cases, and stroke in 8 cases. If compared to Group 1, the mortality rate in Group 2 was significantly reduced (*p* = 0.004). Likewise, the rate of permanent SCI was significantly lower in Group 2 if compared to Group 1 (*p* = 0.023), with an incidence of 7.8% and 11.9% respectively; the overall rate of permanent SCI was 9.5% (105/1107). Among the patients who experienced permanent SCI, 47 patients (44.8%) presented with an early SCI (within 24 h after the surgical procedure), and 58 patients (55.2%) presented with late SCI symptoms. 

The overall incidence of respiratory failure was 29.1% (322/1107), with 31.9% in Group 1, and 27.1% in Group 2; renal failure occurred in 5.8% of the cases, 7.2% in Group 1, and 4.7% in Group 2. Both respiratory failure and renal failure were reduced in the second period (Group 2), but the difference was not statistically significant (*p* = 0.088 and *p* = 0.079, respectively). A slightly positive trend was also noted concerning the incidence of postoperative myocardial ischemia, stroke, mesenteric ischemia, and need for re-intervention, but was not statistically significant. The overall postoperative outcomes, and the specific results of the two Groups are summarized in Table 3.

## 4. Discussion

Thoracoabdominal aneurysms are one of the most complex clinical situations in vascular surgery, and in surgery in general. Patients affected by this disease have a risk of life-threatening rupture if untreated; however, the repair itself carries a substantial risk of postoperative complications such as paraplegia, renal failure, cardiac complications, or even death [1,2,3,4,5,6]. In 2016, Coselli et al. published the largest current series of patients with TAAA treated by means of OSR in a high-volume center; they reported a 7.5% mortality rate, and a composite rate of spinal cord deficit of 9.6%. 

Considering the extension of this aortic disease, the surgical and the anesthesiological approaches in patients with TAAA need to be focused on organ protection. In the last decades, new intra- and perioperative tools have been introduced in high volume centers for TAAA OSR, and a multimodal approach with different adjuncts has progressively evolved to maximize organ protection and reduce the surgical trauma. Thus, we decided to compare the results of the patients treated with or without this multimodal approach, in order to evaluate the role of the adjuncts, and also of the increasing surgical experience, with the aim of presenting an updated point of view of the results of TAAA OSR in an experienced aortic center. 

The two consecutive groups, Group 1 (January 1989–December 2009: 455 pts), and Group 2 (January 2010–December 2022: 652 pts) were created in order to include in the second period the routine use of most of the adjuncts. The two groups were similar for the majority of preoperative characteristics, except for history of smoking and previous aortic surgery (Table 1). In Group 2, a higher number of procedures performed after previous open or endovascular repair was noted, and this may be related to the continuously increasing number of endovascular procedures performed in both the thoracic and the abdominal aortic segments [29,30]. Notably, the progressive employment of different adjuncts in Group 2, and the concomitant introduction of them, makes the evaluation of the effective role of any single adjunct hard to define. Thus, we focused our analysis of the results on the multimodal approach used, with the cumulative inclusion of the different adjuncts (Group 2), more than on the effective role of any single adjunct. The same consideration is valid also concerning the surgical strategy and the introduction of variations in the surgical technique. 

Since 1989, when the first TAAA OSR was performed at our institution, with the “clamp and go” technique, many things have changed from the surgical point of view, but the majority of the modifications have been introduced during the last decade. The surgical strategy was significantly different between Group 1 and Group 2 concerning the site of aortic clamping, the technique used for visceral vessel reconstruction, and the frequency of intercostal artery reattachment (Table 2). Although the TAAA extensions were similar between the two groups, in Group 2, a significantly higher number of “high” proximal clamping between LCCA and LSA was reported (*p* < 0.001). With the development of surgical experience, the aortic neck between the LCCA and the LSA was considered a “safer zone” for proximal cross-clamping, if compared to the aortic portion distal to LSA, and was frequently preferred during extent I–II TAAA OSR. A better aortic quality with a lower risk of wall lesions was observed at this level, and this aortic region was considered the preferred zone for a safer proximal clamping in 16.7% of the cases in Group 2. A trend toward smaller aortic patches was also observed in Group 2, with a significant reduction in the number of Carrel patches with the inclusion of four vessels and an increase in three-vessel Carrel patches associated with selective bypass (*p* < 0.001). This change in the surgical approach was performed after the observation of visceral aortic patch aneurysms during the follow-up, in order to reduce the risk of subsequent dilatation [31,32]. A more aggressive intercostal artery reattachment was also performed in Group 2 (*p* = 0.044); many reasons may help to explain this significant variation between the two groups, among them, the increase in surgical experience, the updated evidence regarding the protective role of this procedure, and the intraoperative use of MEP&SSEP, have played a role and may explain this significant difference between the two groups [18,20,33,34]. 

Concerning the different organ protection strategies, in 2010, we introduced the CoroCTA as a routine part of the preoperative workup. Significantly flow-limiting coronary artery stenosis was addressed before TAAA OSR with percutaneous interventions, using bare-metal stents when possible, or bypass [9]. Since 2013, during the operation, a continuous cardiac evaluation was routinely performed with TEE, with the possibility to apply maneuvers in response to cardiac modifications such as volume loading and vasoactive medications in order to avoid hypotension. Concerning SC protection, one of the most important adjuncts in this field is the CSFD; its protective role during TAAA OSR was first reported in a randomized clinical trial in the early nineties, and it is now well established and also recommended by the most recent guidelines [14,15]. At our institution, in 2003, we started using a drip chamber-based system, and ten years later, in 2013, we introduced in our practice an automated pressure-controlled system, LiquoGuard. This device allows safe and effective automated drainage with lower over-drainage complications compared with the previous system [16]. The CSFD was used significantly more in Group 2 (*p* < 0.001); it has been preoperatively placed in all the patients with extent I–III TAAA treated by means of OSR, without specific contraindications, and only selectively in patients with extent IV, considered at high risk for spinal cord ischemia. However, in accordance with recent evidence, a word of caution needs to be given about CSFD; in fact, it comes with possible complications that should be taken into account [35]. 

LHBP is another essential tool during this surgery, and its protective role in TAAA OSR has been well known since the early 1990s, when we also started to employ this adjunct [36]. This partial external circulation comes with different advantages: it needs low heparinization, and allows both SC and visceral protection, but it also comes with a cardiac protective effect, reducing cardiac afterload during the aortic cross-clamping. 

Intraoperative neuro-monitoring with MEP&SSEP offers another advantage in the complex field of SC protection. It allows continuous intraoperative control of the SC function with the possibility of immediately detecting impairments in SC blood supply and applying adjunctive maneuvers to restore an adequate inflow to the SC, such as: increasing arterial pressure proximally and distally to the clamp with LHBP, early reattachment of hypogastric arteries, and the selective reattachment of critical intercostal arteries [18]. Jacobs’ group reported his initial clinical experience with this technique during TAAA OSR in 1997, and we routinely introduced it into our clinical practice in 2012 [37]. An interesting tool used to reduce the postoperative pain is the intraoperative intercostal nerve cryoanalgesia, and it was introduced in our practice in 2021; in our early experience with this adjunct, it was associated with reduced thoracic pain during the postoperative period, with possible associated respiratory improvements [24,38]. 

Renal failure is one of the most feared complications during and after TAAA OSR, and it is known to be associated with perioperative mortality. For this reason, renal protection strategies have a key role during TAAA OSR, and above all, include perfusion of the kidneys using cold crystalloid solutions [39]. Cold Lactated Ringers is the universally approved solution for kidney perfusion during renal ischemia; however, HTK solution, usually employed for cardioplegia during cardiac surgery, may also be considered a valid alternative for renal perfusion due to its well-known efficacy in preserving kidneys during organ transplantations [21]. We started to use HTK solution for kidney perfusion during TAAA OSR in 2009, and its effective role in preserving the renal function was reported in a prospective, randomized, double blind, clinical trial [40]. Another adjunct introduced during TAAA OSR in Group 2 was the ROTEM, a rapid system able to obtain information about fibrinogen and fibrinolysis during the procedure. A subanalysis of TAAA patients treated by means of OSR between 2009 and 2017 was carried out in order to assess whether the introduction of a ROTEM-based transfusion strategy reduced allogenic blood transfusion and affected morbidity. A propensity score matching was performed to compare patients managed with ROTEM, and patients managed with the previous protocol, based on estimated blood loss and conventional coagulation tests. Patients managed with ROTEM received fewer red blood cell units (3.5 [range 0–11] vs. 4 [range 0–17]; *p* = 0.026) and a lower volume of fresh frozen plasma (286 + 496 vs. 2050 + 1120; *p* < 0.001). In addition, patients in the ROTEM group showed a significant decrease in the occurrence of pulmonary complications (44% vs. 83%; p 1⁄4 0.01) [25]. Lastly, concerning postoperative respiratory failure, which remains the most frequent complication after TAAA OSR, we have been using early prophylactic NIV during the postoperative period since 2014. NIV is able to improve the lung volumes, improve gas exchange, re-open atelectasis, increase ventilation, and reduce the risk of pneumonia. This approach has led to a reduction in postoperative respiratory complications and mortality rate [27]. 

As reported in Table 2, the use of CSFD, TEE, MEP&SSEP, LHBP, renal perfusion with HTK solution and ROTEM, were significantly more used in Group 2. As previously mentioned, TAAA OSR management at our institution has been multimodal in patients included in Group 2, and while the impact of any single adjunct may be hard to define in the entire cohort of patients, the progressive and routine introduction of the different systems has led to significantly better results in terms of mortality (*p* 0.004), and SCI prevention (*p* = 0.023) in our recent experience. The use of this multimodal approach has also led to a reduction in respiratory failure, renal failure and many other postoperative complications, but the impact was not statistically significant. 

In addition, the approach actually used at our center may be considered not only multimodal, but also multisystem; in fact, a single adjunct may play different protective roles; for example, LHBP has a protective SC effect, but also avoids visceral ischemia and cardiac afterload. In the same way, preoperative CoroCTA has a role in preventing cardiac complications, but cardiac problems could led to hemodynamic instability, with possible low perfusion for SC and visceral vessels; these considerations are applicable for the majority of the adjuncts using during TAAA OSR. 

Although the dawn of TAAA endovascular repair is clearly upon us, it is not yet clear whether this approach will benefit all patients or only select groups of them. At this time, also in accordance with the currently available guidelines, TAAA OSR it is not an “old-fashioned” approach but it still needs to be considered a valid treatment option, or even the first treatment option, in patients with long life expectancy, and in patients with genetically triggered aortic disorders [15]. TAAA OSR, in fact, still offers good results in experienced hands, and a lower rate of re-intervention at follow-up, if compared with the endovascular techniques. 

## 5. Limitations

Study limitations include the retrospective design using data from a single center. The retrospective nature of this study also limits the possibility of evaluating other preoperative, intraoperative, and postoperative features that might have influenced the outcomes. In addition, it should be underlined that the time-point chosen to divide the entire cohort into two groups with respect to the adjuncts (Group 1: 1989–2009; Group 2: 2010–2022) was liberally decided by the authors of this paper. The time-point was decided in order to include in Group 2 the more recent experience, and the patients treated with the systematic use of all the currently available adjuncts, in respect of their time of introduction. 

## 6. Conclusions

We have presented our single-center experience with TAAA OSR, which spans approximately three decades. The use of the different adjuncts in a multimodal and multisystem approach enabled us to achieve a significant improvement in mortality and SCI prevention, and an improvement, although not significant, in the rate of other postoperative complications after 2010. Although we have refined our surgical technique and our multimodal approach over time, in an effort to prevent early death and life-altering complications, continued improvements are needed in this complex aortic field. 

## Figures and Tables

**Table 1:** Demographics, and baseline characteristics.

	*Group 1* (455 pts)	*Group 2* (652 pts)	*p*	*Total* (1107 pts)
***Preoperative characteristics***				
Age, years	71.3 (+6.5)	72.1 (+7.2)	0.653	71.8 (+7.0)
Male sex	337 (74.1)	497 (76.2)	0.411	834 (75.3)
Hypertension	286 (62.8)	434 (66.6)	0.203	720 (65.0)
History of smoking	223 (49.0)	274 (42.0)	**0.021**	497 (44.9)
Diabetes	72 (15.8)	87 (13.3)	0.246	159 (14.4)
Dyslipidemia	191 (42.0)	265 (40.6)	0.657	456 (41.2)
History of coronary artery disease	114 (25.1)	176 (27.0)	0.470	290 (26.2)
Cerebrovascular disease	34 (7.5)	33 (5.1)	0.097	67 (6.1)
Chronic obstructive pulmonary disease	55 (12.1)	68 (10.4)	0.387	123 (11.1)
Chronic renal failure	167 (36.7)	253 (38.8)	0.478	420 (37.9)
Aneurysm diameter, mm	64 (+7)	67 (+11)	0.223	66 (+9)
Previous aortic surgery	37 (8.1)	79 (12.1)	**0.033**	116 (10.5)
***Extension***				
TAAA extent I	93 (20.4)	137 (21.0)	0.817	230 (20.8)
TAAA extent II	127 (27.9)	191 (29.3)	0.616	318 (28.7)
TAAA extent III	141 (31.0)	206 (31.6)	0.830	347 (31.3)
TAAA extent IV	94 (20.7)	118 (18.1)	0.286	212 (19.2)
***Etiology***				
Degenerative	328 (72.1)	458 (70.2)	0.506	786 (71.0)
Dissection	96 (21.1)	155 (23.8)	0.295	251 (22.7)
Other	31 (6.8)	39 (6.0)	0.575	70 (6.3)

TAAA: thoracoabdominal aortic aneurysm.

**Table 2:** Intraprocedural details, and intraoperative adjuncts.

	*Group 1* (455 pts)	*Group 2* (652 pts)	*p*
***Intraoperative details***			
CSFD	204 (44.8%)	566 (86.8%)	**<0.001**
TEE	12 (2.6%)	617 (94.6%)	**<0.001**
MEP&SSEP	7 (1.5)	547 (84.0%)	**<0.001**
Procedural time, minutes	258 (201–390)	231 (198–377)	0.063
LHBP	371 (81.5%)	578 (88.7 %)	**<0.001**
Proximal clamping			
Between LCCA and LSA	26 (5.7%)	109 (16.7%)	**<0.001**
Distally to the origin of the LSA	277 (60.9%)	341 (52.3%)	**0.005**
DTA mid/distal portion	152 (33.4%)	202 (31.0%)	0.394
Intercostal artery reattachment	217 (47.7%)	351 (53.8%)	**0.044**
Visceral artery reattachment			
Carrell patch 4-vessels (or beveled)	261 (57.4%)	156 (23.9%)	**<0.001**
Carrell patch (or beveled) + selective bypass	133 (29.2%)	389 (59.7%)	**<0.001**
Selective bypasses	61 (13.4%)	107 (16.4%)	0.170
Renal artery perfusion with HTK solution	73 (16.0%)	557 (85.4%)	**<0.001**
Use of ROTEM	0	198 (30.4%)	**<0.001**

CSFD: cerebrospinal fluid drainage; TEE: trans-esophageal echocardiography; MEP&SSEP: motor and somatosensory evoked potentials; LHBP: left heart bypass; LCCA: left common carotid artery; LSA: left subclavian artery; DTA: descending thoracic aorta; HTK solution: histidine-tryptophan-ketoglutarate solution; ROTEM: rotational thromboelastometry.

**Table 3:** Postoperative outcomes.

	*Group 1* (455 pts) n (%)	*Group 2* (652 pts) n (%)	*p*	*Total* (1107 pts) n (%)
***Perioperative results***				
30-day mortality	61 (13.4)	53 (8.1)	**0.004**	114 (10.3)
Respiratory failure	145 (31.9)	177 (27.1)	0.088	322 (29.1)
Permanent SCI	54 (11.9)	51 (7.8)	**0.023**	105 (9.5)
Renal failure	33 (7.2)	31 (4.7)	0.079	64 (5.8)
Myocardial ischemia	12 (2.6)	9 (1.4)	0.131	21 (2.0)
Stroke	5 (1.1)	8 (1.2)	0.845	13 (1.2)
Mesenteric ischemia	13 (2.9)	14 (2.2)	0.451	27 (2.4)
Need for re-intervention	35 (7.7)	39 (6.0)	0.262	74 (6.7)

SCI: spinal cord ischemia.

## Data Availability

Data unavailable for consultation due to privacy restrictions.
